# Landscape of Tumor Suppressor Mutations in Acute Myeloid Leukemia

**DOI:** 10.3390/jcm9030802

**Published:** 2020-03-16

**Authors:** Cristina Panuzzo, Elisabetta Signorino, Chiara Calabrese, Muhammad Shahzad Ali, Jessica Petiti, Enrico Bracco, Daniela Cilloni

**Affiliations:** 1 Department of Clinical and Biological Sciences, University of Turin, 10124 Turin, Italy; cristina.panuzzo@unito.it (C.P.); elisabetta.signorino@unito.it (E.S.); k.kalabrese81@gmail.com (C.C.); shahzad.sbs@pu.edu.pk (M.S.A.); jessica.petiti@unito.it (J.P.); 2 Department of Oncology, University of Turin, 10124 Turin, Italy; enrico.bracco@unito.it

**Keywords:** acute myeloid leukemia, tumor suppressors, mutations, overall survival, relapse, epigenetic, DNA repair, cell cycle

## Abstract

Acute myeloid leukemia is mainly characterized by a complex and dynamic genomic instability. Next-generation sequencing has significantly improved the ability of diagnostic research to molecularly characterize and stratify patients. This detailed outcome allowed the discovery of new therapeutic targets and predictive biomarkers, which led to develop novel compounds (e.g., IDH 1 and 2 inhibitors), nowadays commonly used for the treatment of adult relapsed or refractory AML. In this review we summarize the most relevant mutations affecting tumor suppressor genes that contribute to the onset and progression of AML pathology. Epigenetic modifications (TET2, IDH1 and IDH2, DNMT3A, ASXL1, WT1, EZH2), DNA repair dysregulation (TP53, NPM1), cell cycle inhibition and deficiency in differentiation (NPM1, CEBPA, TP53 and GATA2) as a consequence of somatic mutations come out as key elements in acute myeloid leukemia and may contribute to relapse and resistance to therapies. Moreover, spliceosomal machinery mutations identified in the last years, even if in a small cohort of acute myeloid leukemia patients, suggested a new opportunity to exploit therapeutically. Targeting these cellular markers will be the main challenge in the near future in an attempt to eradicate leukemia stem cells.

## 1. Introduction

**Figure 1:**
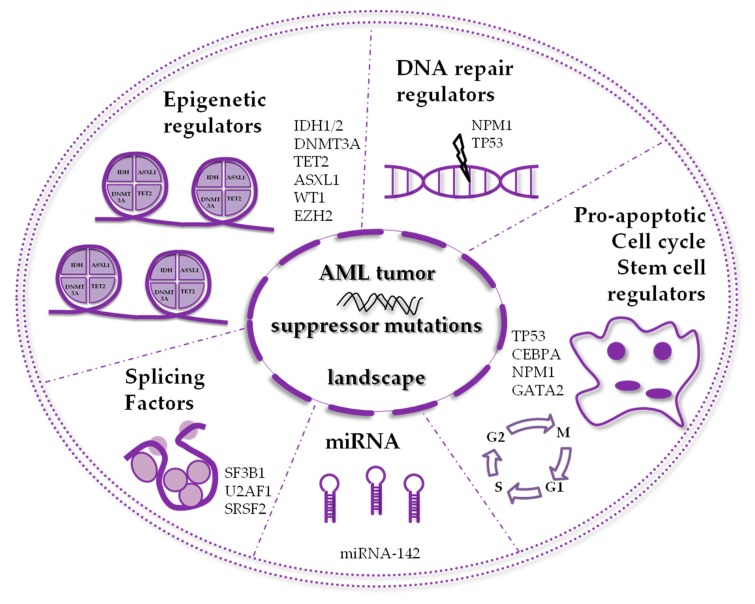
Schematic representation of the frequently mutated tumor suppressor proteins in acute myeloid leukemia (AML).

Acute myeloid leukemia (AML) is the most common acute blood malignancy in adults [1], and it arises as the result of somatically acquired genetic alterations in hematopoietic stem cells (HSCs) [2,3,4]. The incidence of AML increases with age. Though in recent years improvements in therapies led to more favorable prognosis for younger patients, in the elderly the outcome still remains adverse [5]. In most cases AML appears as a de novo disease, but it can also occurs in patients with a previously diagnosed hematological disorder, such as myelodysplastic syndromes (MDSs) or Philadelphia-negative myeloproliferative neoplasms (Ph^−^ MPNs) [6], and in these cases it is usually more resistant to conventional chemotherapy treatments [7]. The pathogenesis of AML shows an excessive proliferation, reduced differentiation and decreased apoptosis of stem cells of myeloid lineage [6,8]. Normal precursors in the bone marrow are replaced with excessively proliferating malignant leukemic cells, leading to hematopoietic failure [8,9]. Leukocytosis and bone marrow failure are common AML clinical signs, whereas infection or bleeding are common cause of death when AML is left untreated [1,10]. There are four frequent translocations in AML, namely PML-RARα, AML1(RUNX1)-ETO(RUNX1T1), CBFα-MYH11 and MLL-fusions, and also other low-incidence oncofusion genes [7]. Furthermore, in the majority of cases, genetic mutations appear without any cytogenetic aberration [11,12]. AML patients are divided into three groups, based on their cytogenetical status: favorable, intermediate and adverse risk (Table 1) [10]. AML was among the first cancers to be studied by innovative microarray and sequencing techniques [13], concluding that AML is a complex disease evolving through time [11,14,15]. In The Cancer Genome Atlas (TCGA) project for AML, several genes such as *FLT3*, *NPM1*, *DNMT3A*, *CEBPA*, *IDH1* and *IDH2*, were found to be recurrently mutated, as well as others never documented before in the pathogenesis of leukemia, including *EZH2* [12]. Some common mutations in AML were found to be directly implicated in the pathogenesis of the disease, being mutually exclusive with all fusion oncogenes involving transcription factors. Moreover, the TCGA project also revealed that in AML the clonal population is indeed heterogeneous, and several subclones may coexist [12]; those clones often bear mutations in genes involved in epigenetic regulation. These observations suggest that they arise as early events, persist over time, survive leukemia chemotherapy and eventually cause relapse [14,15,16,17]. Currently, in routine clinical practice, diagnosis of AML is confirmed with blast count ≥20% on bone marrow smear, immunophenotyping and cytogenetical analysis recognizing chromosomal rearrangements (karyotyping and FISH analysis) combined with molecular analysis of mutated genes, such as *NPM1*, *CEBPA*, *RUNX1*, *FLT3* (both internal tandem duplication (ITD) and tyrosine kinase domain (TDK)), *ASXL1* and *TP53* [10]. Other mutations should be evaluated in case of available clinical trials with new drugs, such specific inhibitors for *IDH1* and *IDH2*, or hypomethylating agents in the presence of *WT1* and *TET2* mutations. In this review, we will outline a picture of the most frequently mutated tumor suppressor genes in AML, such as *IDH1*, *IDH2, TET2, DNMT3A* and *WT1* (Table 2), *NPM1, CEBPA and TP53* (Table 3) (Table 2 and Table 3), as well as others recently discovered to be involved in the disease with a lower mutation frequency, including *EZH2*, *GATA2*, splicing factors and miRNAs (Figure 1).

## 2. AML Mutated Tumor Suppressors Involved in Epigenetic Mechanisms

In this first section, we highlight tumor suppressors commonly mutated in AML, whose function, when mutated, is to deregulate epigenetic mechanisms. 

### 2.1. IDH1 and IDH2 Mutations

**Figure 2:**
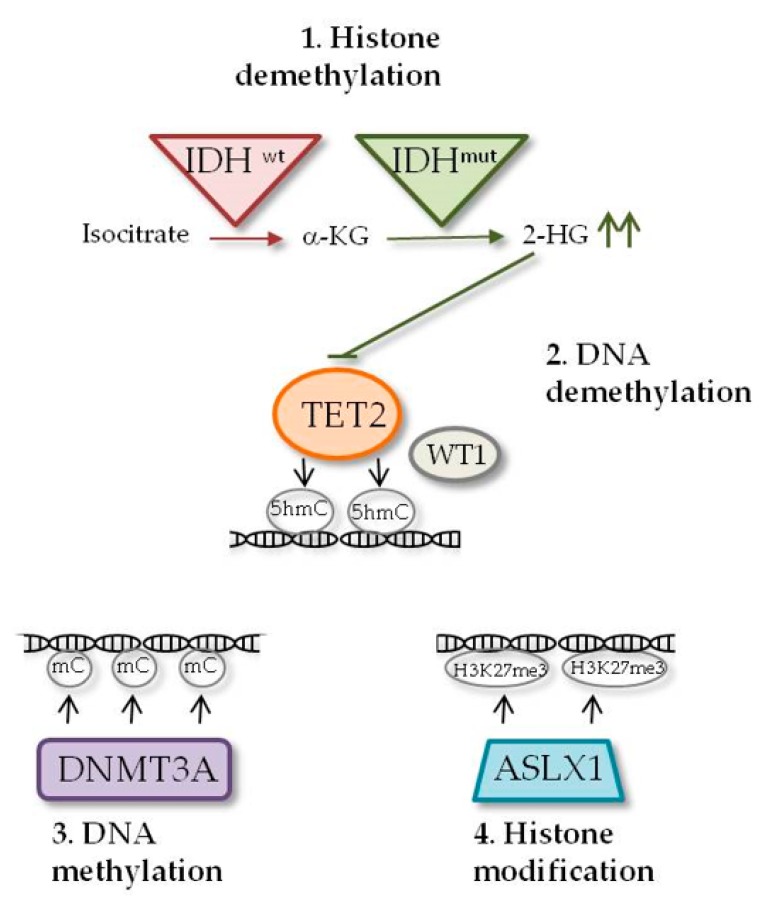
Epigenetic regulators commonly mutated in AML. This scheme highlights the network of proteins involved in epigenetic activity, divided in four epigenetic levels: (**1**) histone demethylation, (**2**) DNA demethylation, (**3**) DNA methylation and (**4**) histone modification. In detail, (**1**) and (**2**) highlight the dynamic interplay between IDH1/2, TET2 and WT1: mutated IDH1 and IDH2 inhibit the activity not only of various histone demethylases, but also of DNA demethylase TET2, through the generation of oncometabolite 2-HG, in turn resulting in DNA hypermethylation; mutated TET2 loses the demethylating activity and causes a hypermethylation profile for itself; mutated WT1is unable to interact with TET2, impairing TET2 demethylating activity. (**3**) Mutated DNMT3A loses the methyltransferase ability, thus resulting in DNA hypomethylation. (**4**) Mutated ASXL1 loses the ability to methylate histone H3 via PRC2 complex, causing a deregulation of key genes involved in stem-cell maintenance and myeloid differentiation. α-KG, α-ketoglutarate; 2-HG, 2-hydroxyglutarate; 5hmC, 5-hydroxymethylcytosine; mC, methylcytosine; H3, histone H3; K27me3, trimethyl at 27th lysine residue.

Isocitrate dehydrogenase (IDH) is an enzyme that catalyzes the oxidative decarboxylation of isocitrate in ketoglutarate (KG), an irreversible reaction of the tricarboxylic acid cycle (TCA). There are three forms, located on three different chromosomes, with different intracellular localization and coenzymes interactions: IDH1 is located within cytoplasm and peroxisomes and is NADP^+^-dependent, while IDH2 and IDH3 are mitochondrial enzymes, with the first being NADP^+^- and the second NAD^+^-dependent [18]. Missense mutations associated with different solid and blood tumors have been identified for *IDH1* and *IDH2*, but not for *IDH3*. These mutations were initially identified in gliomas [19], and later in AML [20], occurring at specific arginine residues within the catalytic active sites of the enzymes: mutations of *IDH1* affect codon R132, with a single amino acid substitution from arginine to histidine, cysteine, serine, glycine, leucine or isoleucine, while mutations of *IDH2* involve residues R140 or R172, where they commonly cause a change from arginine to glutamine or lysine, respectively, but other amino acid substitutions are possible [21]. The result of these mutations is a neomorphic activity of the enzyme that causes the formation of D-2-hydroxyglutarate (2-HG), a metabolite with oncogenic properties [22]. Its accumulation inhibits various α-KG-dependent dioxygenases involved in epigenetic regulation, including those responsible for histones and DNA demethylation, such as TET1/2 methylcytosine hydroxylases (Table 2, Figure 2) [23,24,25]. Consistently, *TET2* inactivation is mutually exclusive with *IDH1* and *IDH2* mutations [26]. The hypermethylation induced by *IDH1* and *IDH2* mutations results in cell differentiation arrest [23]. Rare cases of patients bearing both *IDH1* and *IDH2* mutations have been reported [27]. In AML, *IDH1* and *IDH2* mutations are found in about 10%–30% of patients, with a higher frequency in patients with cytogenetically normal AML (CN-AML) [18]. Prognosis of patients harboring mutations in *IDH1* and *IDH2* is generally poor [7], with an increased probability of relapse [28]. Prognosis could be even worse, with a decreased overall survival (OS), when patients bear other mutations, such as *NPM1*, *FLT3*, *DNMT3A*, *ASXL1*, *RUNX1*, and *NRAS*. For this reason, *IDH1* and *IDH2* mutational status alone is not useful to define prognosis [18]. On the other hand, some studies suggest that *IDH1* and *IDH2* mutations could contribute to progression from MDS or MPN to AML, through a mechanism of reactive oxygen species (ROS) accumulation and DNA damage leading to stabilization and activation of HIF-1 [29,30,31]. Recently, the Food and Drug Administration (FDA) approved IDH1 and IDH2 inhibitors ivosidenib and enasidenib for the treatment of adult relapsed or refractory AML with *IDH1* and *IDH2* mutations [25].

### 2.2. DNMT3A Mutations

The *de novo methyl transferase 3A* (*DNMT3A*) gene encodes for a highly conserved 130-kDa protein involved in epigenetic regulation [32,33]. DNMT3A can be found in the nucleus as dimer, tetramer, or larger structures, and it regulates gene expression through methylation of the cytosine residue of CpG islands [34,35]. Mutations in *DNMT3A* were originally identified in AML patients in 2010 [36] and subsequently in other adult hematological cancers, often arising as early event in AML pathogenesis [17,37,38]. Most of *DNMT3A* mutations found in hematological cancers are located within the methyltransferase domain, with a higher prevalence (about 65%) of heterozygous missense mutations at codon R882 [12,36,38,39,40]. The most common mutation is R882H, that has been proven to act as a dominant-negative on the wild-type *DNMT3A* [41,42,43], losing the ability to form homotetramers [41,43] and thus reducing the methytransferase activity (Table 2, Figure 2). This could explain the DNA hypomethylation observed in patients carrying this type of mutation [12,44,45,46]. *DNMT3A* mutations are found in 15%–30% of patients with de novo AML and are also found in AML evolving from MDS or Ph^−^MPNs [36,38,40,47,48]. Compared to wild-type patients, those carrying R882 mutations are generally diagnosed with CN-AML with myelomonocytic or monocytic blast morphology, with a higher white blood cell (WBC) count and advanced age [39,40,48,49]. *DNMT3A*-mutated AMLs frequently harbor other mutations, such as *NPM1* and *FLT3* mutations [12,39,40]. Prognosis of patients harboring *DNMT3A* R882H mutation seems to be worse than for patients with wild-type *DNMT3A*, although large prospective studies are not available yet. Until then, to define the prognosis of these patients, other validated parameters should be considered, such as age, cytogenetic abnormalities, minimal residual disease (MRD) and presence of other mutations. Furthermore, *DNMT3A*-mutated cells are still present in AML patients with long-lasting complete remission, and this is consistent with the idea that epigenetic mutations, in this case *DNMT3A* mutations, could be preleukemic events, raising the question of whether *DNMT3A* should be used to monitor MRD [17,50,51]. This could also support the idea that additional mutations arising as a second hit in a preleukemic *DNMT3A*-mutated clone could be in some cases responsible for relapse [52].

### 2.3. TET2 Mutations

Ten-eleven translocation-2 (TET2) is a protein involved in epigenetic regulation, as it controls hydroxymethylation by converting 5-methylcytosine to 5-hydroxymethylcytosine, leading to DNA demethylation [53]. TET2 is important during hematopoiesis, as it promotes self-renewal of HSCs, lineage commitment and terminal differentiation of monocytes [54]. Expression of *TET2* gene variants in myeloid cancers was established for the first time in 2009 [55]. *TET2*-inactivating mutations result in a decrease of 5-hydroxymethylcytosine, and this parameter has been proposed as a potential diagnostic and prognostic marker for hematological cancers (Table 2, Figure 2) [56]. *TET2* mutations are very heterogeneous, including frame shifts, nonsense and missense mutations and in-frame deletions, and can be homo- or heterozygous [53]. Both homo- and heterozygous mutations in the *TET2* gene can be found in hematological cancers in patients with similar clinical signs and no difference in OS [57], although patients with homozygous mutations show an inferior event-free survival (EFS) and a higher relapse rate [58]. The frequency of *TET2* mutations in AML patients is about 12%–34% [59]. They occur early during the pathogenesis and could collaborate with other mutations to promote different hematological cancers [53]. *TET2* mutations are associated with CN-AML or intermediate-risk cytogenetic abnormalities and with increased age, higher WBC and blast counts, low platelet count and *FLT3-ITD*, *NPM1* and *ASXL1* mutations, but are mutually exclusive with *IDH1* and *IDH2* mutations [7,53]. Clearly, different combinations of *TET2* and other gene mutations will foresee different outcomes, and the prognostic value of *TET2* mutations remain controversial [60]. Recently, with the introduction in the clinical practice of hypomethylating agents (HMAs), such as azacitidine and decitabine, in adverse-risk-group patients, it seems that the clinical prognosis of patients bearing *TET2* mutations could be improved, since the presence of these mutations could foresee a more favorable response to this type of treatment [53]. 

### 2.4. WT1 Mutations

*Wilms tumor 1* (*WT1*) is a tumor suppressor gene responsible for the development of familiar Wilms’ tumor, from which it takes its name [61,62]. *WT1* gene encodes for a transcription factor that contains four zinc finger motifs at the C-terminal and a DNA-binding domain rich with proline–glutamine at the N-terminal [63]. It is involved in regulation of cell survival, proliferation and differentiation [61,64]. There are four major isoforms of WT1, deriving from two different splicing events: the first causes a 17 amino acid insertion in exon 5 and the second inserts three amino acids at the end of exon 9, leading to a decreased DNA-binding and transcription factor ability and an increased RNA binding [65,66]. Some studies demonstrated that differential expression of isoforms may have a clinical significance in AML [67]. In normal hematopoiesis, *WT1* expression is detectable in CD34^+^CD38^−^ population, while in other populations, *WT1* levels are low, suggesting a role in self-renewal of quiescent cells [68,69]. *WT1* was found overexpressed in AML patients [70], leading to chemotherapy resistance, decreased OS and higher relapse incidence when chemotherapy fails in reducing its expression levels [62,71]. In addition to this oncogenic role, several mutations in *WT1* gene can be found in 6%–15% of de novo AML, including amino acid substitutions, deletions and insertions, and usually occur in exons 1, 7 and 9 [72]. These mutations are frequently nonsense, and the resulting truncated protein can be either expressed or degraded via nonsense-mediated decay [73]. *WT1* mutations are often found in younger patients and correlate with *FLT3-ITD* and *CEBPA* biallelic mutation [72,74]. Analysis of a large cohort of AML patients [11,75] revealed that *WT1* mutations anticorrelate with *TET2* and *IDH1/2* mutations, suggesting that WT1 may have a role in the same epigenetic pathway [76]. Promoter DNA methylation microarrays on the same cohort demonstrated a hypermethylation pattern and 5-hmC levels reduction in patients with *WT1* mutations, a signature very similar to those bearing mutations in *TET2* and *IDH1/2* genes [76], probably due to the ability of WT1 to directly interact with TET2 and TET3. Indeed, *WT1* mutations result in a loss of TET2 function (Table 2, Figure 2) [76,77]. Given the epigenetic alterations due to *WT1* mutations, the use of HMAs such azacitidine is being explored as a potential strategy of therapy in *WT1*-mutated patients [62]. Moreover, *WT1* mutations are usually associated with a negative prognostic outcome and resistance to conventional chemotherapy [78]. Finally, the significance of some polymorphisms has also been investigated, among which the role of SNP rs16754 has been highlighted as a positive prognostic factor in patients with AML [79,80]. 

### 2.5. ASXL1 Mutations

The *additional sex combs-like 1* (*ASXL1*) gene on 20q11 chromosome encodes for a polycomb chromatin-binding protein which acts as an enhancer of the trithorax and polycomb genes [81,82]. It is homolog of the *additional sex combs* (*Asx*) gene of *Drosophila* [83], where it plays a crucial role in embryo development and in the determination of segment identity. ASXL1 acts as an epigenetic scaffold protein by binding to chromatin and recruiting polycomb repressive complex 2 (PRC2), consisting of EZH2, EED and SUZ12 [84]. In this way it regulates the expression pattern of genes involved in both hematopoietic and non-hematopoietic systems [85]. It was firstly identified as a coactivator of retinoic acid receptor (RAR), and among its targets are the *HOX* genes [86]. It is involved in histone modifications, such as histone H3 tri-methylation at 27th lysine residue (H3K27me3) [87], and directly interacts with histone modifiers including NCOA1 (histone acetyltransferase) and LSD1 (histone demethylase) [88]. It was already detected as a component of the PR-DUB complex, related to the deubiquitination of histone H2A [89]. Mice models carrying *ASXL1* mutation showed myeloid dysplasia and shorter survival, mainly due to PRC2 inactivation [90]. Mutations in the *ASXL1* gene have been described in many subtypes of myeloid malignancies and are associated with adverse prognosis, shorter OS and higher risk of progression [88,91]. The frequency is slightly different between single groups. The highest percentage of mutated patients can be found in chronic myelomonocytic leukemia (CMML), followed by myelofibrosis, secondary AML, MDS and de novo AML, with frequencies of about 50%, 35%, 30%, 15% and 8%, respectively [92,93]. Acquired *ASXL1* mutations are frequently frameshift and nonsense, around the Gly-rich domain (amino acids 642-685) on exon 12, and cause the expression of truncated ASXL1, with loss of the PHD domain, crucial for the regulation of key genes involved in stem-cell maintenance and myeloid differentiation (Table 2, Figure 2) [94]. The most common is in position G646. The incidence of *ASXL1* mutations increases significantly with age and correlates with t (8;21), trisomy 8 (+8) and del(7q)/− 7 chromosomal aberrations [95,96]. Otherwise, *ASXL1* mutations are frequently associated with other mutations, such as *RUNX1* and *IDH2*, conferring poor prognosis, far less with *FLT3* and *NPM1* mutants [96,97]. Furthermore, an epigenetic drug screening demonstrated a hypersensitivity of *ASXLl* mutant cells to BET bromodomain inhibitors [98]. Lastly, ASXL1 is one of the fusion protein partners of PAX5 in B-cell acute lymphoblastic leukemias [99].

## 3. AML Mutated Tumor Suppressors Involved in Non-epigenetic Mechanisms

In this second section, we describe other frequently mutated AML tumor suppressors whose function is not involved in epigenetic mechanisms.

### 3.1. NPM1 Mutations

**Figure 3:**
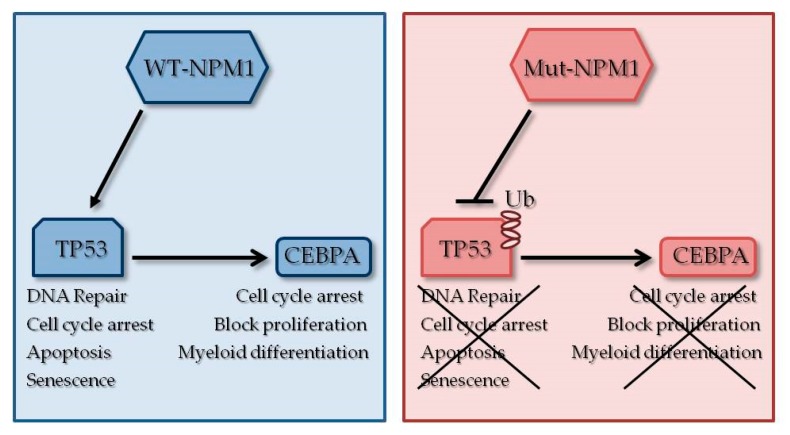
Relevant mutated non-epigenetic tumor suppressors in AML. This scheme highlights proteins’ interaction and pathway deregulation in AML blast with known tumor suppressors mutations. The blue panel shows, in detail, the positive interplay involving wild-type NPM1, increase in TP53 stability (via Mdm2 ubiquitin-ligase activity inhibition) and in CEBPA activity. The red panel shows how, in presence of mutated NPM1, TP53 is highly ubiquitinated, degraded and is unable to activate CEBPA.

The gene *nucleophosmin* (*NPM1*), located on 5q35, encodes a nucleus–cytoplasm shuttling protein [100]. In 2005, an unusual cytosolic localization was identified and associated with the presence of mutations [101]. Functionally, NPM1 is involved in the regulation of several cellular processes such as centrosome duplication [102], DNA repair [103], ribosomal protein assembly and apoptotic response to oncogenic stimuli [103]. NPM1 is a key regulator of tumor suppressors TP53 and p19ARF [104,105], thus contributing to modulate growth-suppressive pathways. Mutations are typically heterozygous and mostly located in exon 12 (Table 3, Figure 3) [106]. They lead to an insertion of four nucleotides determining an open reading frameshift which in turn generates a de novo nuclear export signal [106,107]. As a result, the nucleolar localization signal is lost and the protein relocalizes within the cytoplasm [101]. Furthermore, NPM1 mutants (NPM1c) acquire the ability to impound the wild-type form, preventing the NPM1 wild-type tumor suppressor functions [108]. Mouse models of mutated *NPM1* (*NPM1c*) support the importance of *NPM1c* as a cooperative event in leukemogenesis, but not to initiate leukemia [109]. The impact of *NPM1* mutations on prognosis has been extensively examined over the last decade. They can be found in 25%–30% of AML patients, and their frequency rises in adult AML (near 30%–40%), especially CN-AML [101,110]. Patients with this genotype are classified as favorable risk [111] in the absence of concomitant *FLT3-ITD* mutations, correlating with good response to conventional therapy and high complete remission rates, EFS and/or OS [11,112]. Risk associated to NPM1 mutations deserves a more accurate evaluation when occurring with *FLT3* (fms-related tyrosine kinase 3) mutations. Indeed, co-occurrence of *NPM1* and *FLT3*, either when the latter harbors the more common ITD or the less frequent D835 point mutations, significantly improves the response and the survival outcomes over that of an isolated *FLT3* mutation, thus defining a highly favorable prognostic AML group [113]. Furthermore, the level of *NPM1* mutations is generally used for monitoring MRD [114,115]. Interestingly, the co-occurrence of *NPM1* and *FLT3* mutations is consolidated [112], with a frequency near to twice that of correlation with the wild-type form, suggesting a direct molecular link between them, which has not yet been investigated. This combination is associated with an intermediate prognosis [116]. Moreover, *DNMT3A*, *IDH1*, *IDH2* and *TET2* mutations are identified as concomitant to *NPM1* mutations [12,117], confirming the dynamic interplay among AML tumor suppressors. Finally, *NPM1* may be involved in chromosomal translocations with *ALK* (t(2;5)(p23;q35)), which represents the anaplastic large-cell lymphoma molecular landmark [118] and with *RAR*-*α* (t(5;17)(q35;q21)), causing a subtype of acute promyelocitic leukemia (APL) [119].

### 3.2. CEBPA Mutations 

*CEBPA*, an intronless gene on chromosome 19q31.1, encodes for a zinc finger transcription factor [120], which plays a pivotal role in the differentiation of multipotent precursor cells into myeloid progenitors [121]. In particular, by an advanced interplay between the activation of transcription of myeloid differentiation and inhibition of myeloid proliferation genes, it directs towards granulocyte and monocyte differentiation. CEBPA recognizes the CCAAT sequence on the promoters of target genes and by the interaction with CEBPB and CEBPC activates their functions. The genes directly regulated by CEBPA are divided into four big categories: growth factor receptors, transcription factors (PU.1, c-Jun, c-Myc, SOX4 and E2F), primary and secondary granule proteins and microRNAs (miR-223, miR-34 and miR-30) [122]. Recently CEBPA has been described for its ability to control self-renewal properties of hematopoietic stem and progenitor cells (HSPCs) [123]. Moreover, knockout mice for *CEBPA* or with a mutation in the *CEBPA* basic region displayed a complete block of myeloid differentiation and increased levels of hematopoietic stem cells [124]. The possibility of using two different types of start codons (AUG and an alternative in frame GUG) gives rise to two protein isoforms called p42 and p30 [125]. The second isoform is smaller than the first, without the N-terminal domain that is crucial to promote proliferation arrest by direct inhibition of E2F transcription factors. Moreover p30 exhibits a dominant-negative effect over p42 isoform [125]. The ratio of p30/p42 is critical for a correct granulopoiesis. For this reason, levels of the two isoforms are tightly controlled at the translational level in response to extracellular conditions [126]. Mutations in *CEBPA* gene occur in 5%–20% of de novo AML [127], in both the C and the N terminals of the gene. In-frame insertions or deletions in C-terminal mutations of *CEBPA* disrupt the DNA-binding and homodimerization domains, while out-of-frame insertions or deletions in the N-terminal result in abolishing the translation of full length CEBPA, leading to overexpression of the shorter p30 isoform [126].The peculiarity of these mutations is that they are frequently biallelic, and this feature was associated with favorable prognosis if compared to cases with single allele mutation [128]. Results obtained from gene expression profiling confirmed a peculiar signature associated with biallelic *CEBPA* mutations, therefore the 2016 WHO classification of myeloid neoplasms defined it as a distinct diagnostic entity [129]. Multiple mechanisms for CEBPA inhibition have been identified in leukemic cells, from genetic to epigenetic, from transcriptional to translational and post-translational levels (Table 3, Figure 3). Mutations of *CEBPA* are also associated with mutations in *TET2*, the most frequently co-mutated gene (34%), followed by *GATA2* (21%), *WT1* (13%), *DNMT3A* (9%) and *ASXL1* (9%) [130,131,132,133]. Recently, a direct co-occurrence between *CEBPA* and granulocyte colony-stimulating factor receptor (*CSF3R*) mutations has been reported [134,135]. Notably, near 30% of patients with *CEBPA* biallelic mutations feature a *CSF3R* mutation; in these patients the co-occurrence induces a worsening outcome [136,137].

### 3.3. TP53 Mutations 

*Tumor protein 53* (*TP53*) is a tumor suppressor gene located on chromosome 17p13.1. It regulates cell cycle arrest, apoptosis, senescence and DNA repair. It has been initially described as “the guardian of the genome” referring to its role in preserving genome stability through the prevention of mutations [138]. The encoded protein is characterized by three main domains: an N-terminal transcription-activation domain (TAD), which activates further transcription factors; a central DNA-ligand domain (DNA-binding core domain DBD) enriched in zinc Zn^+^ ions and arginine amino acid residues; and a C-terminal homo-oligomerization domain (OD) [139]. More than 50% of human tumors carry *TP53* mutations, including hematological malignancies, where it has been observed mutated in 5%–20% of AML patients [12]. However, in therapy-related AML and in those with complex karyotype, the rate of *TP53* mutations or deletions increases dramatically (approximately 70%) [140,141]. *TP53* mutations are associated with resistance to chemotherapy, poor OS and poor disease-free survival (DFS) [142,143]. The sharp association with complex karyotype confirms TP53 as a pivotal guard of genome stability [144,145]. TP53 is also involved in the regulation of stem cell quiescence and self-renewal by directly interacting with telomerase [146]. Thus, the malignant clone may benefit from the presence of a TP53 mutated form that accelerates the ability of leukemia stem cells to proliferate after therapy, to accumulate mutations and to become resistant [142,143]. The vast majority of *TP53* mutations occur in the region encoding the DNA-binding domain. Notably, six mutational hot-spots residues were identified R175, G245, R248, R249, R273 and R282, with R273 and R248 being the most frequently mutated [142,147]. Mutations are typically heterozygous, however they are usually followed by a rapid loss of heterozygosity (LOH) due to the high instability of *TP53^+/-^* clones [148]. They are mutually exclusive with other mutated genes (*NPM1*, *FLT3*, *MDM2* and *ARF*) [12,149] but commonly co-occur with del(5q), del(7q) and del(17p) cytogenetic abnormalities [140,141,148,149]. Finally, TP53 pathway may be altered also in presence of wild-type TP53 by several inactivating processes including *MDM2* and *MDMX* overexpression [149,150], miRNA overexpression (e.g., miR-3151, miR-125b) and *FLT3-ITD* mutations, which also promote TP53 inactivation. Additional aberration including *SIRT1* overexpression with subsequent TP53 deacetylation, *CRM1* overexpression and nuclear export of TP53, destruction of TP53 regulator PML in *PML-RARα* positive AMLs and *NPM1* mutations via dysregulation of ARF-MDM2-TP53 axis [150,151,152,153] can affect TP53 pathway (Table 3, Figure 3). The pivotal role of TP53 led to the development of targeted therapies with the aim of reactivating TP53 function. Dual inhibitors of MDM2 and MDMX have been developed and used in clinical trials [150]. Finally, combination therapies with BCL2 inhibitors and TP53 activators might be promising, taking into account the ability of TP53 to regulate MCL-1 degradation [154].

## 4. Other Relevant Mutated Tumor Suppressors

In this last section we will discuss other mutations, with high relevance in leukemogenesis but that occur less frequently in the AML mutational landscape. 

### 4.1. EZH2 Mutations

The *enhancer of zeste 2* (EZH2) gene encodes for a catalytic component of the polycomb repressive complex 2 (PRC2), and its role in epigenetic regulation is enacted through di- and trimethylation of lysine 27 of histone H3, thus leading to transcriptional repression. In normal hematopoiesis, EZH2 is involved in maintaining multipotency and self-renewal of HSCs [84,155]. Low EZH2 protein levels in AML can be due to inactivating mutations in about 2% of adult AML [156], but more often this decrease is dependent on post-translational dysregulation of the protein, triggered by EZH2 phosphorylation induced by CDK1 and subsequent proteasomal degradation. Another mechanism responsible for decreased EZH2 levels in AML is the 7q chromosomal deletion (−7), since *EZH2* gene is located on this chromosome arm and these patients are often resistant to chemotherapy and characterized by a poor prognosis [84,156], Finally, splicing alteration due to mutations in genes involved in the splicing machinery, including *SRSF2* and *U2AF1* (near 10% of AML patients) decreases *EZH2* transcript [155,157]. For this reason, *U2AF1* and inactivating *EZH2* mutations are mutually exclusive [157]. 

### 4.2. Splicing Factors Mutations 

Splicing factors (SFs), notably *SF3B1*, *U2AF1* and *SRSF2*, are well established mutated genes in MDS, with different frequency (45–85%) depending on their subtypes [158,159,160]. The impact on prognosis of these mutations in AML is poorly investigated. Only one study of Hsin-An Hou et al. performed on 500 adult de novo AML outlines near 10% incidence of SFs mutations, all located in hotspot areas [161]. They identified a correlation with intermediate-risk cytogenetics, with *RUNX1*, *ASXL1*, *IDH2* and *TET2* mutations, and they found an association with poor prognosis in term of shorter DFS and OS.

### 4.3. miRNA Mutations

miRNA play a pivotal role in sustaining AML by dysregulating several processes such as proliferation, apoptosis, quiescence and disease progression [162,163]. It is well established that their expression level may affect miRNA functions, making them feasible biomarkers for predicting prognosis [164]. Downregulation of many of them induces changes in DNA methylation (miR-29), cell proliferation (miR-34, miR-146a, miR-223, miR-9), growth (miR-29, miR-139-5p, miR-193a, miR-22), differentiation (miR-223, miR-34, miR-193a, miR-9, miR-22) and eventually apoptotic rate (miR-34c-5p, miR-193a, miR-223) as a result of overexpression of their target genes [165,166,167,168,169]. miRNA nucleotide mutations are relatively rare in AML. In one study by Trissal et al., miRNA 142-3p arises as the only recurrently mutated miRNA in TCGA AML dataset, with a frequency of 2% [170]. These mutations reduce miR-142-3p and miR-142-5p levels and contribute to increase the expression of Hoxa9/a10 target genes in myeloid committed compartment. 

### 4.4. GATA2 Mutations

The *GATA2* gene encodes for a transcription factor involved in the regulation of hematopoietic stem cell activity and self-renewal [171] and in myeloid and erythroid progenitor cell differentiation [172]. *GATA2* mutations are identified in MDS [173] in blast crisis of chronic myeloid leukemia (CML) [174]. In addition, *GATA2* gene mutations are also found in de novo AML [175,176], being mainly concentrated within the exon 3 which encodes for the zinc finger domain 1, with a frequency near to 4%, which rises to 12% in the FAB M4 subtype [177]. Interestingly, *GATA2* mutations are often associated with *CEBPA* biallelic mutations and, with lower incidence, to *NPM1* and *FLT3* mutations [176,178]. Furthermore, patients harboring both *CEBPA* biallelic and *GATA2* mutations show a more favorable prognosis and better OS than those with *CEBPA* biallelic mutations alone [175]. Curiously, germline *GATA2* mutations frequently occur in Emberger syndrome, in monocytopenia and mycobacterial infection (MonoMAC) and in secondary AML [179,180,181]. 

## 5. Conclusions

The genetic heterogeneity of AML patients and the coexistence of multiple subclones are usually the most common cause of relapse. Nowadays, nearly 50% of AML patients relapse after the first cycle of induction chemotherapy. Additional genetic changes might arise, thus leading to the selection of novel resistant subclones. Furthermore, due to their plasticity, subclones can easily adapt and escape standard treatments. The accurate identification of mutated genes is currently considered important for patients’ stratification and, as a consequence, for therapeutic decisions. In recent years many efforts were addressed to ascertain AML potential targets associated with either resistance to therapy or disease relapse. With the advent of mass-spectrometry-based methods performed directly on human AML-sorted stem cells, a significant number of leukemia-specific proteins, especially membrane-associated, have been identified. The main objective of this approach has been the identification of novel AML stem cell biomarkers to exploit as immunotherapeutic targets, in order to eradicate the disease [182,183,184,185]. Moreover, patients’ proteomic profiles could correlate with the mutational status and thus with the prognosis of AML patients, suggesting that proteogenomic approaches might become the main goal in the near future. In terms of next-generation sequencing (NGS), the establishing of an accurate genetic profile at the onset of the disease has allowed designing tailored therapies aiming to eradicate residual mutated clones. In clinical practice, the detection of tumor suppressor gene mutations is performed not only for the diagnosis but also to control and measure MRD. Indeed, the risk of relapse is sharply related to the persistence of MRD after chemotherapy. Gene mutation profile has affected not only the prognosis, as in the case of the co-occurrence of *NPM1* and *FLT3*, but also the choice of treatment, since some of them become therapeutic targets (e.g., IDH1/2, WT1 and TET2) (Table 4). In addition, some epigenetic regulators (DNMT3A, TET2, ASXL1) come out as age-related mutated genes in healthy elderly subjects, an event known as age-related clonal hematopoiesis. Therefore, they have become relevant to predict the onset of hematologic malignancies but not to monitor the MRD. In conclusion, further studies are still needed in order to explore the dynamic interplay between tumor suppressors, oncogenes and persistence of mutations, to help clarify patients’ classification and determine who might benefit from additional therapeutic strategies.

## Figures and Tables

**Table 1:** Cytogenetic and molecular profile of prognostic-risk groups.

Prognostic-Risk Group	Cytogenetic Aberrations and Molecular Abnormalities
Favorable	t(8:21)(q22;q22) *AML1(RUNX1)-ETO(RUNX1T1)* inv(16)(p13;1q22)*CBFα-MYH11* t(15;17)(q22;q12)*PML-RARα* *NPM1* mutation without *FLT3*-ITD or with *FLT3*-ITD^low^ * CEBPA biallelic mutations
Intermediate	*NPM1* mutation with *FLT3*-ITD^high^ * NPM1 wild-type without *FLT3*-ITD or with *FLT3*-ITD^low^ * (in the absence of adverse risk genetic lesions) t(9;11)(p22;q23)*MLLT3-KMT2A* Other cytogenetic abnormalities not included in the other groups
Adverse	t(6;9)(p23;q34)*DEK/NUP214* inv(3)(q21;q26.2)*GATA2,MECOM(EVI1)* t(9;22)(q34.1;q11.2)*BCR-ABL1* t(v;11q23.3)*KMT2A(MLL)* rearranged −5 or del(5q) −7 or del(7q) abn(17p) Complex karyotype Monosomal karyotype *NPM1* wild-type and *FLT3*-ITD^high^ * RUNX1 mutations (in the absence of favorable risk genetic lesions) *ASXL1* mutations (in the absence of favorable risk genetic lesions) *TP53* mutations

* Low, low allelic ratio (<0.5); * high, high allelic ratio (≥0.5).

**Table 2:** Summary and features of AML mutated tumor suppressors involved in epigenetic mechanisms.

Mutated Tumor Suppressors Involved in Epigenetic Regulation		
Mutated Genes	Frequency in AML (%)	Functions, Associations, Prognostic Impact and Specific Drugs
*IDH1*	6–10	Enzyme involved in TCA cycle Important role in lipid metabolism Involved in cellular defense of oxidative damage Mutations cause D-2-hydroxyglutarate (D2HG) accumulation that inhibits various dioxygenases involved in epigenetic regulation Frequent in CN-AML Associated with *NPM1* mutations Associated with *FLT3*, *DNMT3A*, *ASXL1*, *RUNX1*, *NRAS* mutations Mutually exclusive with *TET2* mutations Associated with clonal hematopoiesis in healthy elderly persons Early event in leukemogenesis Prognostic impact context-dependent IDH1 inhibitor ivosidenib approved by FDA
*IDH2^R140^*	5–15	Enzyme involved in TCA cycle Involved in cellular defense of oxidative damage Mutations cause D-2-hydroxyglutarate (D2HG) accumulation that inhibits various dioxygenases involved in epigenetic regulation Frequent in CN-AML Frequency increases with age Associated with *NPM1* mutations Associated with *FLT3*, *DNMT3A*, *ASXL1*, *RUNX1*, *NRAS* mutations Mutually exclusive with *TET2* mutations Associated with clonal hematopoiesis in healthy elderly persons Early event in leukemogenesis Prognostic impact could be more favorable than other IDH mutations IDH2 inhibitor enasidenib approved by FDA
*IDH2^R172^*	1–4	Enzyme involved in TCA cycle Involved in cellular defense of oxidative damage Mutations cause D-2-hydroxyglutarate (D2HG) accumulation that inhibits various dioxygenases involved in epigenetic regulation Frequent in CN-AML AML with *IDH2^R172^* mutation (in the absence of other lesions) may represent a separate disease class, associated with a distinct microarray gene expression and microRNA expression profile Mutually exclusive with *NPM1* mutations Associated with *FLT3*, *DNMT3A*, *ASXL1*, *RUNX1*, *NRAS* mutations Mutually exclusive with *TET2* mutations No consistent data on prognostic impact Associated with clonal hematopoiesis in healthy elderly persons Early event in leukemogenesis IDH2 inhibitor enasidenib approved by FDA
*DNMT3A*	15–30	Catalyzes the addition of a methyl group to the cytosine residue of CpG dinucleotides Essential for de novo DNA methylation and regulation of gene expression Frequent in CN-AML Frequency increases with age Associated with *NPM1*, *FLT3-ITD*, *IDH1*, *IDH2^R140^* and *IDH2^R172^* mutation Prognostic impact not consistent and context-dependent Particularly poor prognosis in *DNMT3Amut/NPM1mut/FLT3-ITD* Persistent *DNMT3A* transcript levels in hematologic CR Associated with clonal hematopoiesis in healthy elderly persons Early event in leukemogenesis
*TET2*	12–34	Regulates differentiation or proliferation and epigenetic modifications Key family of enzymes for DNA demethylation Frequent in CN-AML Frequency increases with age Associated with NPM1 mutation Mutually exclusive with *IDH1* and *IDH2* mutations Prognostic impact associated with inferior OS in CN-AML Associated with clonal hematopoiesis in healthy elderly persons Early event in leukemogenesis Mutations in *TET2* may respond to hypomethylating agents (HMAs) therapy
*WT1*	6–15	Zinc finger transcription factor Multiple isoforms from two splicing events Involved in regulation of cell survival, proliferation, and differentiation Overexpressed in AML where it is used as a diagnostic molecular marker and for MRD monitoring Overexpression correlate with chemotherapy resistance, decreased OS and higher relapse rate Mutations in exons 1, 7 and 9 in AML Frequent in younger patients Associated with *FLT3-ITD* and *CEBPA* biallelic mutation Associated with worse prognosis and resistance to chemotherapy Possible role in the same epigenetic pathway of TET2 and IDH1/2 Anticorrelated with *TET2*, *IDH1* and *IDH2* mutations Use of HMAs such azacitidine as a potential strategy of therapy in *WT1* mutated patients Polymorphism SNP rs16754 positive prognostic factor in patients with AML
*ASXL1*	5–18	Chromatin-binding protein, epigenetic scaffold protein Enhancer of the trithorax and polycomb genes Mutations in the *ASXL1* described in many subtypes of myeloid malignances Associated with adverse prognosis, shorter OS and higher risk of progression Frequent in CMML Frequency increases significantly with age Correlate with t(8; 21), +8 and − 7 chromosomal aberrations Associated with *RUNX1* and *IDH2* mutations Associated with clonal hematopoiesis in healthy elderly persons Early event in leukemogenesis

**Table 3:** Summary and features of AML mutated tumor suppressors involved in non-epigenetic mechanisms.

Mutated Tumor Suppressors Involved in Non-Epigenetic Mechanisms		
Mutated Genes	Frequency in AML (%)	Functions, Associations, Prognostic Impact and Specific Drugs
NPM1	25–30	Nucleus-cytoplasm shuttling protein Involved in the regulation of centrosome duplication, DNA repair, ribosomal protein assembly and apoptotic response to oncogenic stimuli Key regulator of tumor suppressors TP53 and p19ARF Frequent in adult CN-AML Mutations mostly located into exon 12 Correlates with good response to conventional therapy Classified as favorable risk, high complete remission rates, EFS and OS Co-occurrence with *FLT3* mutation associated with an intermediate prognosis Associated with *DNMT3A*, *IDH1*, *IDH2* and *TET2* mutations Used for monitoring of MRD
CEBPA (biallelic)	5–20	Zinc finger transcription factor Regulates differentiation of multipotent precursor cells to myeloid progenitors Directs granulocyte and monocyte differentiation Controls self-renewal properties of hematopoietic stem and progenitor cells Frequent *in* de novo AML Frequently biallelic Biallelic mutations are associated with favorable prognosis if compared to single allele mutation AML subgroup with *CEBPA* mutations recognized as a distinct diagnostic entity by the 2016 WHO classification of myeloid neoplasms Direct transcriptional repression by AML1-ETO, RARα-PLZF and FLT-ITD Associated with *TET2*, *GATA2*, *WT1*, *DNMT3A* and *ASXL1* mutations Associated with a more favorable prognosis
TP53	5–20	Guardian of the genome Regulates cell cycle arrest, apoptosis, senescence and DNA repair Mutation frequency rises in therapy-related and complex karyotype AML (approximately 70%) Mutations associated with absence of clinical remission, poor OS and DFS Majority of mutations in the region encoding the DNA-binding domain Mutations typically heterozygous followed by a rapid loss of heterozygosity Mutually exclusive with *NPM1*, *FLT3*, *MDM2* and *ARF* Associated with -5, -7, -17 cytogenetic abnormalities In presence of wild-type form, several inactivating processes including *MDM2* and *MDMX* overexpression, miRNA overexpression, *FLT3-ITD* mutations and impact on TP53 pathway Targeted therapy influenced by low frequency mutations Therapy focused on reactivate the wild-type TP53 Dual inhibitors of MDM2 and MDMX in clinical trials in AML Combination therapies with BCL2 inhibitors (venetoclax)

**Table 4:** Summary of the impact of tumor suppressor genes mutation on prognosis and recommendations for clinical testing.

Mutated Gene	Prognosis		Current Diagnostic Practice ^1^
*ASXL1*	Poor	Worse OS Correlation with age > 60 years and higher WBC counts	Recommended by 2017 ELN guidelines
*CEBPA*	Variable	Positive in CN-AML Biallelic mutations have better EFS, DFS and OS Single mutations with *NPM1*mut/*FLT3*-ITD^low^ cases have worse OS compared with *CEBPA* wild-type *NPM1*mut/*FLT3*-ITD^low^ cases Impaired outcome with concurrent *TET2* mutation Better OS with concurrent *GATA2* mutation	Recommended by 2017 ELN guidelines
*DNMT3A*	Poor	Linked to adverse outcomes	Recommended: pre-leukemic event, could indicate higher probability of relapse
*IDH1*	Not consistent data	Impaired outcome in R132 mut/*FLT3* wild-type patients	Recommended: new specific inhibitor (ivosidenib) in clinical trials
*IDH2*	Not consistent data	R172 showed no correlation to outcome or response R140 improved OS and decreased response rates	Recommended: new specific inhibitor (enasidenib) in clinical trials
*NPM1*	Good	Improved OS, DFS, and relapse-free survival (RFS)	Recommended by 2017 ELN guidelines
*TET2*	Not consistent data	Impaired OS in multivariate analysis Impaired DFS	Recommended: could respond to HMAs treatment
*WT1*	Poor	Often concurrent with *FLT3* mutations Impaired OS and RFS	Recommended: could respond to HMAs treatment
*TP53*	Poor	Associated with resistance to chemotherapy Impaired OS and DFS Association with complex karyotype	Recommended by 2017 ELN guidelines

^1^ Testing for molecular alterations according to the 2017 ELN recommendations.
